# Head and neck cancer: searching for genomic and epigenetic biomarkers in body fluids – the state of art

**DOI:** 10.1186/s13039-019-0447-z

**Published:** 2019-07-11

**Authors:** Ilda Patrícia Ribeiro, Joana Barbosa de Melo, Isabel Marques Carreira

**Affiliations:** 1 Cytogenetics and Genomics Laboratory, Faculty of Medicine, University of Coimbra, Pólo Ciências da Saúde, Coimbra, Portugal; 2 iCBR-CIMAGO - Center of Investigation on Environment Genetics and Oncobiology - Faculty of Medicine, University of Coimbra, Coimbra, Portugal

**Keywords:** Body fluids, Cell-free DNA, Circulating tumor DNA, Exosomes, Head and neck cancer

## Abstract

Head and neck squamous cell carcinoma (HNSCC) affects multiple sites of the upper aerodigestive tract and exhibited high incidence and mortality worldwide, being frequently diagnosed at advanced stage. Early detection of HNSCC plays a crucial role in a successful therapy. In the last years, the survival rates of these tumors have not improved significantly due to the late diagnosis and the lack of precise disease biomarkers and targeted therapies. The introduction in the clinical practice of body fluids to detect and analyze circulating tumor cells (CTCs), circulating tumor DNA (ctDNA) and exosomes provides a minimally or non-invasive method also called as liquid biopsy for diagnostic and prognostic biomarkers detection, representing a shift of paradigm in precision medicine through the revolution in the way to perform HNSCC diagnosis and to screen high risk population. Despite the use of body fluids being an emergent and up-to date issue to early diagnosis HNSCC and their recurrences, no strategy has yet proven to be consistently effective and able to be translated to clinical application in the routine clinical management of these patients. In this review we will discuss the recent discoveries using blood and saliva to identify biomarkers for the early detection and prognosis of HNSCC.

## Introduction

Head and neck squamous cell carcinoma (HNSCC) is the sixth most common cancer worldwide with an annual incidence of around 600 000 new cases, mostly diagnosed as locally advanced disease [1]. This carcinoma is a heterogeneous disease at clinical and molecular level, encompassing several tumors from hypopharynx, oropharynx, lip, oral cavity, nasopharynx, and larynx. This tumors group presents different epidemiology, etiology and molecular alterations that drive carcinogenesis and, consequently distinct therapy responses. The traditional risk factors related to the pathogenesis of HNSCC are smoking and excessive alcohol comsumption, being also infection with high-risk human papillomaviruses (HPVs) associated to a rising number of these tumours, especially at the oropharynx in younger patients [2]. Human papillomavirus-related oropharyngeal cancer (HPV+) exhibited not only better response to treatment but also better survival, being generally associated to a good prognosis when compared to HPV-negative [3, 4], which lead to the adaptation of the eighth edition of the HNSCC tumour-node-metastasis (TNM) staging in order to include p16^INK4A^ immunostaining as a surrogate for HPV status. The HPV-positive cancer incidence is rising, while HPV-negative cancers incidence is decreasing [5]. The five-year overall survival rate of HNSCC patients is almost unchanged in the last decades, remaining around 50%, even with the improvements in the treatment (i.e surgery, radiotherapy, chemotherapy and novel targeted therapies), mainly due to the advanced clinical tumor stage at the diagnosis and the treatment failure associated to frequent recurrencies [6]. The HNSCC treatment selection is based in some clinical-pathological parameters, such as the tumor anatomic location and tumor stage; however, these patients with similar clinic-pathological characteristics may differ in their clinical outcome, justifying the tumor’s biologic heterogeneity [7]. A better understanding of the molecular biology of HNSCC is pivotal to shed light in the body of knowledge of HNSCC with practical implications in the patients’ management and in the HNSCC precision medicine. The relatively recent advent of high throughput omics technologies and consequently the multi-level molecular integrative approaches is a great promise to stratify HNSCC patients in subgroups according to their molecular and clinical profiles, identifying diagnosis and prognosis biomarkers, for the selection of adequate drug targets and also the design of personalized treatment strategies. Moreover, non invasive screening programs targeting these biomarkers in body fluids of high-risk individuals like smokers, excessive alcohol consumers as well as patients during and after treatment for monitoring residual disease and relapses or metastases could improved early detection with sucessfull curative interventions and quality of life preservation [7].

In this review, we focus on recent findings based on body fluids approaches to identify and detect biomarkers and how they are providing clues to change the paradigm of HNSCC patients management.

### Field cancerization and genomic alterations in head and neck Cancer

Head and Neck Cancer results from multiple cumulative epigenetic and genetic alterations that sequentially lead to transformation of a normal cell into a neoplastic cell. The field cancerization concept was first introduced in 1953 by Slaughter et al. [8], describing histologically abnormal tissue surrounding oral squamous cell carcinoma, which may explained the frequent development and high incidence of multiple primary tumors and locally recurrent cancer in the HPV-negative patients, since in these HPV-positive patients the role of field cancerization concept need to be clarified. The advent of genomic era can help to reveal the genetic basis of these transformed fields and the identification of biological progression models in which the development of a field with genetically altered cells plays a central role in the multistep selective carcinogenesis process [9]. The first genetic model of HNSCC was described by Califano et al. [10], in which losses at chromosomal regions 3p, 9p and 17p were considered early events in the carcinogenic process. However, the molecular alterations may occur before phenotypic ones, reinforcing the great promise of the molecular alterations identification to early detect HNSCC, to monitor potential malignant lesions and to predict the disease progression and behavior. The methodological evolution from Conventional Cytogenetics to array Comparative Genomic Hybridization (array-CGH), Single Nucleotide Polymorphism array (SNP-array) and Next-Generation Sequencing (NGS) has been resulted in systematic efforts to characterize the numerical and structural genomic alterations and mutational spectrum of HNSCC. Alterations in almost all chromosomes have been described in these tumors, being some chromosomal regions and genes reported in the literature as more consistently altered [7], such as copy number gains at 3q, 6p, 8q, 11q, 16p, 16q, 17p, 17q and 19q, and copy number losses at 2q, 3p, 4q, 5q, 8p, 9p, 11q and 18q [7, 11–17]. Moreover, several signaling pathways and key pathway components are known to be disrupted in HNSCC, such as epidermal growth factor receptor (EGFR) signaling, phosphatidylinositol-3-kinase/protein kinase B (PI3-K/Akt) signal transduction pathway, mammalian target of rapamycin (mTOR), nuclear factor–kB (NF-kB) transcription factors and heat shock protein 90 (Hsp90, 18]. Besides this growing HNSCC molecular knowledge, the clinical care of the patients is still almost absent of molecular diagnostic, being the targeted therapy options limited to cetuximab and in the daily clinical practice, the molecular tests have almost no impact in the prognosis and in the prediction of response to therapy [18]. This scenery could be revolutionized by the integration of molecular and clinic-patological data into the diagnostics and treatment process as well as with the use of circulating biomarkers to monitor patients and risk populations.

### Circulating tumor biomarkers

Tissue biopsies frequently do not reflect tumor heterogeneity and behavior, being multi-site biopsies repeated sequentially unpractical and, in some rare cases, even one single biopsy cannot be performed, such as the cervical lymph node metastasis of squamous cell carcinoma with occult primary. A promising alternative to overcome these problems is the liquid biopsy, a less invasive method to monitor the real-time dynamics of cancer [19]. So, identification of specific biomarkers in circulation represents a promising strategy to track tumor-specific alterations during the course of the disease and during the monitoring process of high-risk populations, being the presence of circulating tumor DNA (ctDNA), circulating tumor cells (CTCs) or also the analysis of circulating exosomes and microvesicles, possible indicators for disease recurrence or lack of response to treatment.

Circulating tumor DNA (ctDNA) is cell free DNA (cfDNA) that is shed from tumor cells into the circulatory system, carrying somatic mutations from primary and/or secondary tumors and representing only a small fraction (< 1.0%) of total cfDNA [20, 21]. ctDNA seems to result from tumor deposits and lysed CTCs in circulation, however, its origin is yet uncertain [20]. Nowadays, the question if ctDNA has an active role in carcinogenesis or whether it is a byproduct of tumor shedding remains to be clarified [22]. ctDNA could be detected in several other body fluids besides blood, such as urine, stools, cerebrospinal fluid, and saliva [23].

Circulating tumor cells (CTCs) are present in bloodstream during the formation and growth of tumors, in low concentration and result from metastatic precursor cells of lymphovasculature released into circulation by primary or metastatic tumors. The CTCs can be enriched and detected through distinct technologies that take advantage of their physical and biological properties [24]. In the last years, the methods for isolating CTCs have evolved and its detection in several cancer types have shown correlations with tumor staging and with patient prognosis [20]. CTCs present as major advantages: i) the possibility to obtain CTC lines to drug sensitivity testing and ii) yield information on a cellular level, demonstrating cell-to-cell variability (clonality) [25]. However, ctDNA analysis could become an alternative to CTCs due to technological difficulties in the isolation and its identification and enrichment among millions of normal hematogenous cells, which require sophisticated equipments; nevertheless ctDNA fragments are diluted with huge amounts of cfDNA from normal cells, which can also be a limitation for further molecular analysis [25].

CTCs and ctDNA seem to share common somatic mutations, genomic rearrangements, epigenetic and protein patterns with the primary and/or secondary tumors and metastases, opening the window for a real-time monitoring of the cancer patients without the need of an invasive tissue biopsy.

Additionally, exosomes and microvesicles have also been found in blood and saliva of cancer patients, namely in head and neck cancer. Exosomes and microvesicles represent the two major subtypes of extracellular vesicles, with different morphology, biophysical characteristics and biogenesis [26]. Exosomes are small membrane vesicles with diameters ranging from 40 to 150 nm with proteins, lipids, RNA, and DNA and a role as promoter of tumour progression or antitumor function [27–29]. Microvesicles are larger in size than exosomes with diameters ranging from 100 to 1000 nm and a heterogeneous and dynamic molecular composition; however, there are no established molecular biomarkers able to distinguish these two classes of vesicles [30]. There are several protocols optimized to purify exosomes and microvesicles from body fluids or cell culture supernatants, being isolated from healthy and diseased individuals in urine, semen, saliva, amniotic fluid, cerebrospinal fluid, lymph, bile, ascites, tears, breast milk, and blood [31–33]. Cancer-cell-derived exosomes seem to be able to modify tumor cell movement and consequently metastasis [34].

Besides the increased number of studies using body fluids, nowadays for HNSCC there are no validated cost-effective non invasive tests to early detect this carcinoma. There are several studies using different body fluids-based tests for detection of circulation biomarkers under investigation; however, the use of these body fluids as liquid biopsy to screening, diagnostic and prognostic approaches require not only sensitive and specific technologies but also complex bioinformatics algorithms. The body fluids most frequently described to detect HNSCC biomarkers are peripheral blood and saliva. However, urine cfDNA can also be used as liquid biopsy for urological and non-urological tumors, since it harbors information on DNA from cells exfoliated in urine and from circulation; nevertheless, its potential application is understudied in non-urological tumors, namely in HNSCC [35].

### Body fluids for detection of circulation HNSCC biomarkers

#### Peripheral blood

A blood test is a minimally invasive approach that can be repeated at different time points during patient’s treatment and follow-up, being a source for the retrieval of DNA and RNA to detect circulating molecular markers. It holds the promise to improve diagnosis, treatment monitoring and surveillance in cancer [36]. The peripheral blood, both plasma and serum, presents circulating nucleic acids, serving as liquid biopsy with diagnostic and monitoring applications. Nowadays, there are several studies using different approaches of liquid biopsy in HNSCC. Total ctDNA concentration independently of ctDNA genomic and epigenetic analysis could be used as a diagnostic and prognostic tool; however, the importance of increased ctDNA in cancer patients remains debatable [22]. Mazurek et al. [37] analyzed in plasma the cfDNA level of 200 HNSCC patients and verified a higher level (*p* = 0.011) of the total cfDNA in oropharyngeal squamous cell carcinoma patients in comparison with other HNSCC. Moreover, the level of cfDNA in the patients with clinical regional lymph nodes N2-N3 tumors was (*p* = 0.015) higher than in the patients with a clinical regional lymph nodes N0-N1 as well as with stage IV compared with stages I-III of cancer (*p* = 0.011). Likewise, the frequency of positive HNSCC CTCs detection seems to be TNM (tumor, node, metastasis) stage dependent. Kawada et al. [38] detected and quantified CTCs in 32 HNSCC patients using a low-pressure filtration system equipped with precision microfilters and verified that patients with advanced disease had higher number of CTCs, but the clinical N (degree of regional lymph node involvement) classification was not related with its quantification. Detection of CTCs in the peripheral blood is associated with a worse prognosis of cancer, with several distinct CTC quantification tests being used in different cancer types, including in HNSCC with an unclear significance [38]. Nichols et al. [39] detected CTCs in 6 of 15 patients with advanced-stage HNSCC using the CellSearch® system, approved by the U.S. Food and Drug Administration for monitoring CTCs in other cancer types, and verified that CTCs levels were significantly associated with lung nodules patients > 1 cm (*p* = 0.04) and also suggested survival improvement in the CTC-negative versus the CTC-positive patients (*p* = 0.11). HNSCC is characterized by some early genetic and epigenetic alterations, being several of these biomarkers explored in circulation. Methylation of *CDKN2A, MGMT, GSTP1,* and *DAPK1* was assayed in HNSCC tissue and serum, being *DAPK1* methylation correlated with lymph node metastasis (*p* = 0.014) and advanced disease (*p* = 0.016), [40]. Schröck et al. [41] demonstrated that quantitative *SEPT9* and *SHOX2* DNA methylation levels in cfDNA from plasma could be biomarkers for diagnosis, molecular staging, prognosis and post-therapeutic monitoring of HNSCC patients.

Several miRNAs in circulation are also under investigation as diagnostic and prognostic HNSCC biomarkers. Since miRNAs are abundant cfRNA molecules in blood and seem to be associated with the solid tumors from which they originate [42, 43]. Hsu et al. [44] verified the expression profiles of ten miRNAs, let-7a, miR-21, miR26b, miR-34c, miR-99a, miR-133a, miR-137, miR-184, miR-194a, and miR-375, in plasma from 50 HNSCC patients using real-time quantitative polymerase chain reaction (PCR) and suggested that detection of circulating miR-21 and miR-26b pre- and post-operatively could be a marker of HNSCC prognosis, being these levels reduced post-operatively in patients with good prognosis. In patients with oral cancer and potential malignant lesions, plasma miR-196a and miR-196b levels were significantly higher than in controls, being the combined detection of these miRNAs as potential biomarker for early detection of oral cancer [45]. Up-regulation of miR-181 was detected in both tumor tissues and plasma and was associated with progression of leukoplakia to invasive oral cancer as well as with lymph-node metastasis, vascular invasion, and poor survival [46].

Likewise, the presence of microvesicles in serum of HNSCC patients has been described, being the origin of these vesicles unclear. Tumor-derived circulating extracellular vesicles presenting in HNSCC serum patients seems to play a role in tumor evasion from cell death, being responsible for the demise of activated CD8^+^ T cells in the peripheral circulation, possibly through the presence of FasL (member of the tumor necrosis factor (TNF) Family) on the vesicles [47]. Further investigation of the molecular content of microvesicles and exosomes seems to be advantageous to identify biomarkers for HNSCC tumor progression and outcome.

Van Ginkel et al. [48] proposed a workflow for application of liquid biopsy in the locoregional surveillance of HNSCC patients following curative treatment. However, further studies using the same methodology to identify, quantify and molecularly analyzed the ctDNA and/or CTCs in larger HNSCC cohorts are needed. It is vital to perform validation studies in different HNSCC cohorts to translate these potential biomarkers for routine clinical application with a clear benefit to patients monitoring and high-risk populations.

#### Saliva

Saliva is the most popular body fluid under investigation to detect oral cancer, presenting the following advantages: accessibility in a non-invasive way, low contamination of normal material (cells, DNA, RNA, and proteins) and inhibitory substances and also less complex in comparasion to blood [49]. Additionally, the use of the saliva fluid phase could be more advantageous than the exfoliated cells use, since there are tumor locations in the head and neck region that are not able to be easily accessed to perform a swab. Especially in oral cancer, saliva samples is considered very important to search early biomarkers due to direct contact with potential malignant and malignant lesions. There are different methods of saliva collection with or without stimuli that may affect the concentrations of analytes present in saliva. Although the non-invasiveness characteristic of saliva, the salivary diagnostics is recognized for oral diseases but its clinical application for systemic diseases is still unclear [50].

Interestingly, the sensitivity of tumor-derived DNA detection in saliva seems to be site-dependent, being most efficient for the oral cavity tumors, whereas plasma seems to be preferentially enriched in tumor DNA from the other sites of the head and neck region [51]. Perdomo et al. [52] found a low concordance of *TP53* mutations detection between HNSCC tumor, oral rinses (11%) and plasma (2.7%) samples. Sethi et al. [53] identified in saliva a discrete genetic signature, comprising genetic alterations in *PMAIP1* and *PTPN1* genes that differentiated HNSCC patients from normal controls. Spafford et al. [54] used microsatellite analysis to detect tumor-specific genetic alterations in exfoliated oral mucosal cell samples from HNSCC patients and verified that microsatellite instability was detectable in saliva of 24 (96%) out 25 cases in which it was present the tumor.

Considering the epigenetic field, altered promoter hypermethylation patterns have been detected in body fluids and exfoliated cells of HNSCC. Ovchinnikov et al. [55] using methylation-specific polymerase chain reaction (MSP) assay identified a three genes panel, *RASSF1A*, *DAPK1*, and *CDKN2A* genes, with ability to detect tumor presence with an overall accuracy of 81% in the DNA isolated from saliva of HNSCC patients when compared with the DNA isolated from the saliva of healthy nonsmoker controls, proving the application of saliva for assessing the hypermethylation status of tumor-suppressor genes. Righini et al. [56] analyzed 90 HNSCC patients and observed a good agreement between methylation of *TIMP3, ECAD, p16, MGMT, DAPK,* and *RASSF1* in tumors and paired saliva samples. Moreover, 22 patients were followed after treatment and hypermethylation was detectable in the saliva of five patients few months before clinical and 2-deoxy-2[18F]fluoro-d-glucose-positron emission tomography signs of relapse and the other 17 patients (16 in remission and 1 relapsed) exhibited a negative result in salivary rinses.

Moreover, exosomes isolation from saliva has also been tested and optimized; nevertheless, the biological mechanisms and functional role in HNSCC saliva-derived vesicles remain unclear [30]. It is believed that the presence of pathologies could influence exosome characteristics, e.g. head and neck cancer saliva-derived vesicles present variations in size, density, and CD63 expression in comparasion to noncancer [30]. There is a lack of data regarding the importance and role of these salivary vesicles and their composition in the prognosis and diagnosis of these tumors.

Despite of the intense research in the saliva using high-troughput technologies, no single molecule or combined putative biomarkers has been shown to have high accuracy and specificity to perform early diagnostics, prognostics, patients monitoring and treatment response. Integration and validation studies are pivotal to evaluate the clinical application of the potential circulating salivary biomarkers described for HNSCC. It is also needed to take in mind that radiotherapy is commonly used for the treatment of HNSCC, being xerostomia one of its most important side effects, which depicts the importance of combine different body fluids in the monitoring of this neoplasm.

## Conclusions

Liquid biopsy can be a minimal or non-invasive tool to molecular profile tumors, with great potential to help in early diagnosis, prognosis, surveillance and treatment monitoring of cancer. Liquid biopsies applications in HNSCC have emerged and suffer a great development in the last years; however, a long walk to validate its application in clinical practice is needed for a significative impact in the patients life. Firstly, the accurate identification and successful application of prognostic biomarkers in HNSCC are very scarce, being emergent the predictive models development of disease evolution and outcome and molecular signatures with clinical impact. Secondly, most of the body fluids studies are performed in small cohorts of patients and there is also a great discrepancy in the methodological protocols used to identify, quantify and analysed the cfDNA and CTCs in blood, saliva and exosomes. Validation studies in larger multi-centers HNSCC cohorts are needed before specific biomarkers being selected and body fluids protocols to be translate to HNSCC clinical practice. It is expected that the molecular and bioinformatic technical improvements, namely the digital advent and massively parallel sequencing techniques, even at the single-cell level will increase the sensitivity and accuracy of multiple biomarkers detection. The early detection of HNSCCs and their relapses will have a beneficial impact in the survival and life quality of these patients but mostly in high-risk populations.

## Data Availability

Not applicable.
